# The 1% Rule in Four Digital Health Social Networks: An Observational Study

**DOI:** 10.2196/jmir.2966

**Published:** 2014-02-04

**Authors:** Trevor van Mierlo

**Affiliations:** ^1^Research AssociateHenley Business SchoolUniversity of ReadingGreenlands, Henley-on-ThamesUnited Kingdom; ^2^Evolution Health Systems IncToronto, ONCanada

**Keywords:** social networks, Superusers, eHealth, 1% rule, Pareto Principal, 90-9-1 principle, moderated support

## Abstract

**Background:**

In recent years, cyberculture has informally reported a phenomenon named the 1% rule, or 90-9-1 principle, which seeks to explain participatory patterns and network effects within Internet communities. The rule states that 90% of actors observe and do not participate, 9% contribute sparingly, and 1% of actors create the vast majority of new content. This 90%, 9%, and 1% are also known as Lurkers, Contributors, and Superusers, respectively. To date, very little empirical research has been conducted to verify the 1% rule.

**Objective:**

The 1% rule is widely accepted in digital marketing. Our goal was to determine if the 1% rule applies to moderated Digital Health Social Networks (DHSNs) designed to facilitate behavior change.

**Methods:**

To help gain insight into participatory patterns, descriptive data were extracted from four long-standing DHSNs: the AlcoholHelpCenter, DepressionCenter, PanicCenter, and StopSmokingCenter sites.

**Results:**

During the study period, 63,990 actors created 578,349 posts. Less than 25% of actors made one or more posts. The applicability of the 1% rule was confirmed as Lurkers, Contributors, and Superusers accounted for a weighted average of 1.3% (n=4668), 24.0% (n=88,732), and 74.7% (n=276,034) of content.

**Conclusions:**

The 1% rule was consistent across the four DHSNs. As social network sustainability requires fresh content and timely interactions, these results are important for organizations actively promoting and managing Internet communities. Superusers generate the vast majority of traffic and create value, so their recruitment and retention is imperative for long-term success. Although Lurkers may benefit from observing interactions between Superusers and Contributors, they generate limited or no network value. The results of this study indicate that DHSNs may be optimized to produce network effects, positive externalities, and bandwagon effects. Further research in the development and expansion of DHSNs is required.

## Introduction

### Background

Research examining digital health social networks (DHSNs) and their feasibility to improve health began in the mid-1980s [1,2]. As these networks became increasingly available, studies focused on relationships between network size, structure, program sustainability [3-5], and motivations of participants [6].

Terminology also developed to define common roles and behavior. For example, members of social networking sites (SNS) are now commonly referred to as actors [7]. Lurking (or passively reading social network conversations without actively participating) is the most common behavior [8]. Conversely, common to SNS are actors who frequently generate content and facilitate discussions [9-12]. In practice, these actors are often referred to as Superusers [13]. The impact or value that Lurkers, Superusers, or other actors have within DHSNs has not been empirically examined.

### Network Effects and Positive Network Externalities

To frame their value, it may be beneficial to view Lurkers, Contributors, and Superusers and other actors through the lens of sociology, political science, economics, and finance where there is a rich history of examining network effects. A network effect occurs when an individual’s use of a good or service influences its perceived value [14,15].

An example of a network effect can be seen in the popularity and growth of the fax machine. When few organizations had fax machines, the value of having a fax machine was low. However, as more organizations purchased fax machines and quickly and efficiently communicated with other departments or organizations, the network of fax machines grew and so did the value of owning one. Over time, having a fax machine in the workplace became essential. This is also known as the bandwagon effect, where the demand for a good increases because others are consuming it [16].

In the above example, the addition of each fax machine created a positive externality [17]. Positive externalities contribute to growth and popularity of a product or good, and social science research is now beginning to investigate this phenomenon within SNS [18].

Introducing the concept of network effects and positive externalities can help explain the importance of recruiting, retaining, and managing different types of actors to help grow DHSNs. If growing a DHSN increases program efficacy, it is important to understand the mechanisms behind content generation and how to increase network effects.

### The 1% Rule (90-9-1 Principle)

Mirroring the well-established Pareto Principle, also known as the 80-20 rule [19], cyberculture and digital marketing have informally adopted a phenomenon named the 1% rule, or 90-9-1 principle [20,21]. Following the principals of a power law, the Pareto Principle is a natural observation illustrating that roughly 80% of effects come from 20% of causes [22]. Similarly, the 90-9-1 principle states that 90% of SNS actors observe and do not participate, 9% contribute sparingly, and 1% create the vast majority of new content. This 90%, 9%, and 1% are also known as Lurkers, Contributors, and Superusers. To date very little empirical research has been conducted to verify the 1% rule.

The purpose of this study was to examine if the 1% rule applied to moderated DHSNs designed to facilitate behavior change. Paid employees who were trained in social cognitive theory [23], motivational interviewing [24], the stages of change [25], and cognitive behavioral therapy (CBT) [26] actively moderated the four DHSNs in this study. Moderator roles focused on facilitating discussions, encouraging problem solving among members, administering compliance with privacy protection rules, protecting the community from spam, and ensuring that all discussions focused on adherence to behavior-change principles.

Furthermore, if DHSNs are efficacious, is it possible to create network effects to increase wellness on a population level? If size of the network matters, how are positive externalities created? More importantly, how do different actors interact and is it possible to create bandwagon effects?

## Methods

### Settings and Program Descriptions

To verify the 1% rule, this observational study analyzed descriptive data from four eHealth interventions that contain large social networks. The four Internet interventions are AlcoholHelpCenter (problem drinking) [27], DepressionCenter (depression) [28], PanicCenter (panic) [29], and StopSmokingCenter (smoking cessation) [30].

All four DHSNs are online, free to participants, do not offer advertising, do not promote any products, and are a part of Evolution Health Systems Inc’s (EHS) social business model. EHS is a private, research-based organization that builds evidence-based digital programs designed to increase medication and treatment adherence. The four DHSNs analyzed in this study were originally built by EHS for research purposes.

During the study period, moderators consistently monitored each DHSN, reviewed all 578,349 DHSN posts, and checked for their accuracy and consistency. Posts that did not specifically address behavior change or comply with program rules were removed.

Full descriptions of each intervention appear elsewhere [31-34]. The oldest of the four DHSNs was nearly 11 years in operation at time of this study, and functionality of each DHSN has been enhanced over time. For explanatory purposes, Table 1 outlines the main features of each program.

Retrospective data were extracted from each program’s structured query language (SQL) database. Descriptive statistics were analyzed in SPSS version 19 for Mac.

All data collection procedures adhered to international privacy guidelines [35-37] and were in accordance with the Helsinki Declaration of 1975, as revised in 2008 [38]. The study was consistent with the University Research Ethics Committee procedures at Henley Business School, University of Reading, and was exempt from full review.

**Table 1:** Program features and functionality.

	Problem drinking	Depression	Panic disorder	Smoking cessation
Moderated social network	✓	✓	✓	✓
Tailored behavior-change program	✓	✓	✓	✓
Brief intervention/screener	✓	✓	✓	✓
Blogs	✓	✓	✓	✓
Private messaging among members	−	✓	−	✓
Video testimonials	✓	✓	✓	✓
Public profile	✓	✓	✓	✓
Symptom diary/tracker	✓	✓	✓	✓
Gamification (techniques to increase usability leveraging desire for achievement, rewards, and competition)	−	✓	−	✓

### Registrants and Study Duration

The four DHSNs had varying numbers of members and life spans (see Table 2). Periods of analysis ranged from 4.0 years (problem drinking) to 10.9 years (smoking cessation).

The dataset was purged of moderator accounts to ensure that all content originated only from registered members. Only registered members could actively contribute to discussions; however, registration was not required to read or review all existing or newly generated content.

**Table 2:** Subjects and study duration.

	Problem drinking	Depression	Panic disorder	Smoking cessation
Date of first post	July 25, 2008	April 5, 2003	January 7, 2002	September 17, 2001
Date of last post	August 7, 2012	August 5, 2012	August 7, 2012	August 7, 2012
Number of days	1474	3411	3866	3978
Years	4.0	9.3	10.6	10.9
Registrants, n	2597	5151	11,372	44,870

## Results

### Summary

Descriptive statistics revealed that less than 25% of actors in each DHSN authored one or more posts (see Table 3).

**Figure 1:**
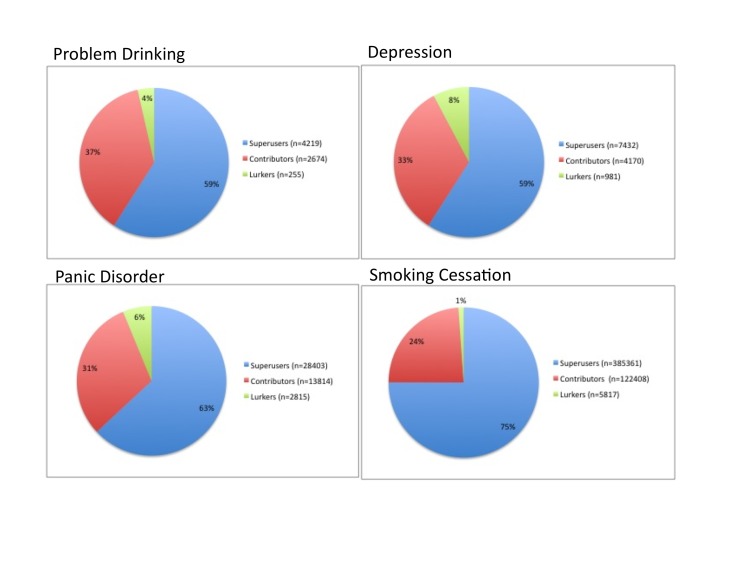
Network content according to the 1% rule.

Post frequencies in each of the four DHSNs were divided into the top 1% (Superusers), the next 9% (Contributors), and the remaining 90% (Lurkers) of actors. Each DHSN revealed similar patterns, with Superusers generating 59.0%-75.0%, Contributors authoring 23.8%-37.4%, and Lurkers only creating 1.1%-7.8% of all posts (see Figure 1 and Table 4).

**Table 3:** Number and percentage of actors making one or more posts.

	Problem drinking	Depression	Panic disorder	Smoking cessation	Total	Mean	Weighted mean
Total actors	2597	5151	11,372	44,870	63,990	15,998	31,934
Actors who made at least one post, n (%)	449 (17.3)	1230 (23.9)	2767 (24.3)	7963 (17.7)	12409 (19.4)	3102 (19.4)	6193 (19.4)

**Table 4:** Analysis of the 1% rule.

	Problem drinking	Depression	Panic disorder	Smoking cessation	Total	Mean	Weighted mean
Total population (n)	2597	5151	11,372	44,870	63,990	15,998	40,875
Total social network posts	7148	12,583	45,032	513,586	578,349	144,587	369,434
1% of population (Superusers)	26	52	114	449	641	160	415
Total posts by Superusers	4219	7,432	28,403	385,361	425,415	106,354	276,034
Percentage of posts by Superusers, %	59.0	59.1	63.1	75.0	73.6	73.6	74.7
9% of population (Contributors)	234	464	1023	4038	5759	2880	3572
Total posts by Contributors	2674	4,170	13,814	122,408	143,066	35,767	88,732
Percentage of posts by Contributors, %	37.4	33.1	30.7	23.8	24.7	24.7	24.0
90% of population (Lurkers)	2337	4636	10,235	40,383	57,590	14,398	27,246
Total posts by Lurkers	255	981	2815	5817	9868	2467	4668
Percentage of posts by Lurkers, %	3.6	7.8	6.3	1.1	1.7	1.7	1.3

### The 1% (Superusers)

On average, the top 1% (n=160) of Superusers created 73.6% (n=106,354) of posts. On an individual program level, the top 1% varied in their overall contributions, but in all cases accounted for the majority of activity, with a weighted average of posts being 74.7% (n=276,034).

### The Next 9% (Contributors)

The second highest group of contributors, or the next 9% of the population, accounted for an average of 24.7% (n=35,767), with a weighted average of 24.0% (n=88,732) of posts.

### The Remaining 90% (Lurkers)

The remaining 90% of the population accounted for an average of 1.7% (n=2467) of posts, with a weighted average of 1.3% (4668) of posts.

### Cumulative Participation

**Figure 2:**
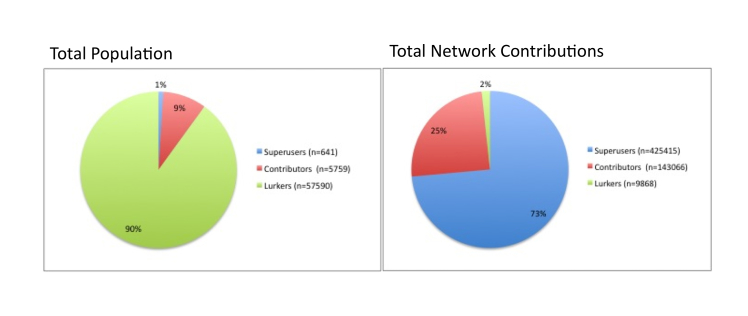
Cumulative DHSN population distribution and content creation according to the 1% rule.

Cumulatively, Lurkers accounted for the vast majority of the population in the four DHSNs (n=57,590); however, this population created only 1.7% (n=9868) posts. Conversely, Superusers accounted for a small amount of actors (n=641) but created 73.6% (n=425,415) posts (see Figure 2).

Based on the overwhelming creation of content from a small number of Superusers and underwhelming amount of number of posts from a large number of lurkers, cyberculture’s 1% rule applies to the creation of positive network externalities in the four DHSNs analyzed in this study.

## Discussion

### Principal Findings

Superusers accounted for a weighted average of 74.7% of content and generated the vast majority of posts within the four DHSNs. These findings match criteria of the 1% rule and may be comparable to the Pareto Principle.

Conversely, Lurkers generated limited or no network value. Although Lurkers may benefit from observing interactions between Superusers and Contributors, they do not generate network effects nor do they contribute to the network growth.

In regards to Superuser participation, motivations and posting patterns in the DHSNs have been previously examined. A 2008 analysis of the problem drinking DHSN found that common themes included introductions, greetings, general supportive statements, suggested strategies, success stories, and discussion of difficulties [9]. In addition, this study found that the amount of discussions varied over time and clustered around nodes consisting of one or more Superusers. A 2010 publication on the smoking cessation DHSN found that the majority of first posts were from recent quitters who were struggling with their quit attempts. Responses were rapid and from seasoned quitters, indicating that the social network may be particularly beneficial for peer support to help relapse prevention [11].

Content analysis has also been conducted on the four DHSNs. A 2009 academic presentation found that a high proportion of first posts in the panic disorder DHSN resembled “panic stories”, suggesting that the network may act as an expressive writing forum [39]. A 2010 academic presentation on the same community found that the support group was used more often by those reporting greater intensity of panic symptoms, absenteeism from work, and that Lurkers completed a greater number of the program’s CBT treatment sessions compared to Contributors and Superusers [40]. A recent University of Toronto PhD dissertation found that depression DHSN users generally sought informational support, various types of emotional support, coaching support, and social companionship [41]. Future research should focus on possible differences between post frequencies and content themes that may be prevalent in different indications, disease states, or actor types.

Based on the observations in this study, health care organizations should focus efforts on recruiting and retaining Superusers. Superusers may have a wide range of options to focus their participation, whether on health-related social networks or those of general interest. Moreover, they may exhibit different patterns of network behavior in different communities [42]. The motivations, needs, and participatory patterns of Lurkers and Contributors should also be examined. Future research should focus on the demographic and psychographic characteristics of these three actor-types.

It is also important to consider that the actions of some Superusers may result in negative network externalities. This type of behavior may result in negative network effects and decrease the size of the network. Conversely, Superusers may generate positive network effects in digital resources that are negatively oriented towards health, promoting illness, or disease [43].

An increasing number of health care organizations are making digital health care tools available to their patients, policyholders, or consumers, and many of them contain social networks. While some DHSNs flourish, many suffer from little or no traffic [44]. Strategies increasing Superuser and Contributor participation can increase the effectiveness of these programs.

A successful DHSN requires active managers who not only guide discussions but also facilitate growth [45]. The findings from this paper indicated that managers of DHSNs should identify Superusers early, encourage their participation, and target their recruitment though offline initiatives. Managers should not expend resources on promoting engagement with Lurkers.

### Strengths and Limitations

A strength of this study is that the four DHSNs have never been promoted or advertised as they are not commercial entities. Participants in the four programs in this study could find the DHSNs only through extensive search efforts, links from other websites, or word-of-mouth. Profit-driven commercial entities focus considerable efforts and budgets on recruitment and promotion (free trials, banner advertising, celebrity endorsement, offline promotion, and other incentives) and most likely attract much larger populations with different motivations [46]. As a result of non-promotion, the four DHSNs in this study may have attracted only naturalistic, self-seeking health populations.

However, lack of advertising or promotion may also be a limitation. The naturalistic self-seeking population of actors within these networks may not be representative of populations that are typically reached from well-promoted programs. Many organizations or trials have promotional or recruitment budgets, thus casting a wider net and attracting a variety of health populations.

Especially in a climate of limited budgets and funding, the influence of promotion or non-promotion should encourage organizations with DHSNs to carefully consider the role of advertising and recruitment, and if those efforts should be strategically targeted.

Finally, only data from registered users were examined. Any visitor could browse the DHSNs without registering, but it is not possible to reliably examine this data nor combine it with the behavior of registered users.

### Conclusions

The 1% rule was consistent across the four DHSNs. However, as individuals can lurk without registering, the 1% (Superusers) may represent an even smaller population. As social network sustainability requires fresh content and timely interactions, these results are important for organizations actively promoting and managing DHSNs.

Superusers generate the vast majority of traffic and create value, so their recruitment and retention is imperative for long-term success. Although Lurkers may benefit from observing interactions between Superusers and Contributors, they generate limited or no network value.

The results of this study indicate that DHSNs have the potential to be optimized to produce network effects, positive externalities, and bandwagon effects. Further research in the development, expansion, and management policies of DHSNs is required.

## Abbreviations

AHC: AlcoholHelpCenter.net
CBT: cognitive behavioral therapy
DC: DepressionCenter.net
DHSN: digital health social networks
PC: PanicCenter.net
SNS: social networking sites
SSC: StopSmokingCenter.net
SQL: structured query language
